# Coal-Packed Methane Biofilter for Mitigation of Green House Gas Emissions from Coal Mine Ventilation Air

**DOI:** 10.1371/journal.pone.0094641

**Published:** 2014-04-17

**Authors:** Hendy Limbri, Cindy Gunawan, Torsten Thomas, Andrew Smith, Jason Scott, Bettina Rosche

**Affiliations:** 1 School of Chemical Engineering, The University of New South Wales, Sydney, Australia; 2 School of Biotechnology and Biomolecular Sciences, The University of New South Wales, Sydney, Australia; 3 Centre for Marine Bio-Innovation, The University of New South Wales, Sydney, Australia; University of Kansas, United States of America

## Abstract

Methane emitted by coal mine ventilation air (MVA) is a significant greenhouse gas. A mitigation strategy is the oxidation of methane to carbon dioxide, which is approximately twenty-one times less effective at global warming than methane on a mass-basis. The low non-combustible methane concentrations at high MVA flow rates call for a catalytic strategy of oxidation. A laboratory-scale coal-packed biofilter was designed and partially removed methane from humidified air at flow rates between 0.2 and 2.4 L min^−1^ at 30°C with nutrient solution added every three days. Methane oxidation was catalysed by a complex community of naturally-occurring microorganisms, with the most abundant member being identified by 16S rRNA gene sequence as belonging to the methanotrophic genus *Methylocystis*. Additional inoculation with a laboratory-grown culture of *Methylosinus sporium*, as investigated in a parallel run, only enhanced methane consumption during the initial 12 weeks. The greatest level of methane removal of 27.2±0.66 g methane m^−3^ empty bed h^−1^ was attained for the non-inoculated system, which was equivalent to removing 19.7±2.9% methane from an inlet concentration of 1% v/v at an inlet gas flow rate of 1.6 L min^−1^ (2.4 min empty bed residence time). These results show that low-cost coal packing holds promising potential as a suitable growth surface and contains methanotrophic microorganisms for the catalytic oxidative removal of methane.

## Introduction

To prevent explosions during underground coal mining, mine shafts are continuously ventilated to dilute methane released from the coal seam to non-combustible concentrations (usually ≤1% v/v since the lower flammable limit of methane is 5% (v/v) in air). The mine ventilation air (MVA) is then released untreated into the atmosphere causing significant greenhouse gas emissions. Methane has an approximately twenty-one times higher potential impact on global warming than carbon dioxide (on a mass-basis in a 100-year time frame) arising from its higher molar absorption coefficient for infrared radiation and a longer residence time in the atmosphere [1]. On a molecular basis this equals a 7.6-times higher impact of methane. Worldwide emissions of methane from coal mining are extensive, estimated to be over 329 million tonnes (Mt) carbon dioxide-equivalent in 2005 [2] with approximately 70% of these methane emissions released as MVA [3].

Major challenges for MVA methane mitigation are the low methane concentrations limiting its usefulness as an energy source, the high flow rates (50–500 m^3^s^−1^) and the considerable variability of these parameters [4]. Various thermal technologies have been considered [5]–[7] and while capable of treating MVA, they attract high capital and operating costs and require considerable safety measures.

Biofiltration technology is a safer and less expensive approach as it utilises microorganisms as biocatalysts to oxidise methane to carbon dioxide and biomass at ambient temperature. Methane-oxidising organisms (methanotrophs) occur ubiquitously and actively grow in environments where both methane and either oxygen or alternative electron acceptors are present (e.g. soils, lakes, ponds, landfills, and coal mine sites). The biology of various methanotrophs has been recently reviewed [8].

Over the past decade, methane biofiltration technology has garnered considerable attention for the treatment of effluent gases generated during landfill and animal husbandry operations, where reasonably low gas flow rates occur [9]–[13]. However, only limited insights are available regarding the potential of biofiltration for the removal of methane at the very low concentrations and high flow rates of MVA (for a recent review see [14]). Studies of methane removal with packing materials such as polypropylene Raschig rings ([15], a batch study), glass tubes [16], mature compost [9], gravel [13], [17] or pine bark [18] revealed relatively slow conversion. Consequently, large biofilter volumes may be required for MVA applications.

To accommodate large volume biofilters, inexpensive packing materials would be required. At a mine site the most convenient and inexpensive packing material may be coal. Chakravorty and Forrester (1985) have reported that methanotrophic microorganisms were able to grow on the surface of coal as a thin biofilm and oxidized methane in batch experiments [19]. To our current knowledge, coal has not been utilised previously as a biofilter packing material for microbial methane oxidation.

The aim of the present study was to design a laboratory-scale continuous biofilter with coal as the packing material for methane oxidation at 1% (v/v) – a concentration representative of MVA - in order to evaluate whether non-sterilised coal may serve as a suitable alternative to other packing materials. Specific objectives were to monitor biofilter performance over a range of gas flow rates and to investigate the effect of inoculation with the methanotrophic bacterium, *Methylosinus sporium*. At the end of the experiment, the composition of the mixed biofilm community was analysed to determine the dominant methanotrophic organism at that time.

## Material and Methods

### Nitrate mineral salts (NMS) medium, coal packing and gases

The nitrate mineral salts (NMS) medium according to the German Resource Centre for Biological Material (DSMZ) contained per litre: 1 g MgSO_4_.7H_2_O; 0.2 g CaCl_2_.6H_2_O; 0.004 g Fe(III)NH_4_-EDTA; 1 g KNO_3_; 0.272 g KH_2_PO_4_; 0.717 g Na_2_HPO_4_.12H_2_O. This medium was mixed with 1 ml L^-1^ methanol (as additional carbon source to accelerate the slow growth of methanotrophs) and 0.5 ml L^-1^ trace element solution. The trace element solution contained (per L): 0.5 g Na_2_-EDTA; 0.2 g FeSO_4_.7H_2_O; 0.01 g ZnSO_4_.7H_2_O; 0.003 g MnCl_2_.4H_2_O; 0.03 g H_3_BO_3_; 0.02 g CoCl_2_.6H_2_O; 0.001 g CaCl_2_.2H_2_O; 0.002 g NiCl_2_.6H_2_O; 0.003 g Na_2_MoO_4_.2H_2_O. The pH of the medium was adjusted to 6.8 with 2 M NaOH. It was then sterilized by autoclaving for 15 minutes at 15 psi and 121°C. Bituminous coal, for use as the biofilter packing material, was kindly provided by BHP Billiton (www.bhpbilliton.com) from the Appin Colliery site in New South Wales, Australia. Coal characteristics are available in the supplementary section Data S1. The coal was ground using a mortar and pestle and sieved to obtain pieces of 2–3 cm diameter. Methane with a purity of 99.95%, argon with a purity of 99.996% and compressed air were obtained from Core Gas, Australia.

### *Methylosinus sporium* inoculum

*Methylosinus sporium* (DSM17706) was obtained from DSMZ (Germany). The species was selected due to its ability to oxidise methane in a low nitrogen environment (so as to minimise the requirement for nutrient supply) [20], [21]. *M. sporium* cultures were grown in sealed 500 mL Erlenmeyer flasks containing 100 mL of NMS medium. After inoculation with 5 mL of stock culture, 8 mL of methane was injected with a syringe through the gas seal of the flask. Flasks were incubated at 30°C and 180 rpm in an orbital shaker and growth was monitored by optical density measurements at a wavelength of 600 nm. Whenever the methane concentration fell below 1% (v/v), 4 mL of methane was added until no further growth was evident after 5 days.

### Biofilter design and operation

**Figure 1:**
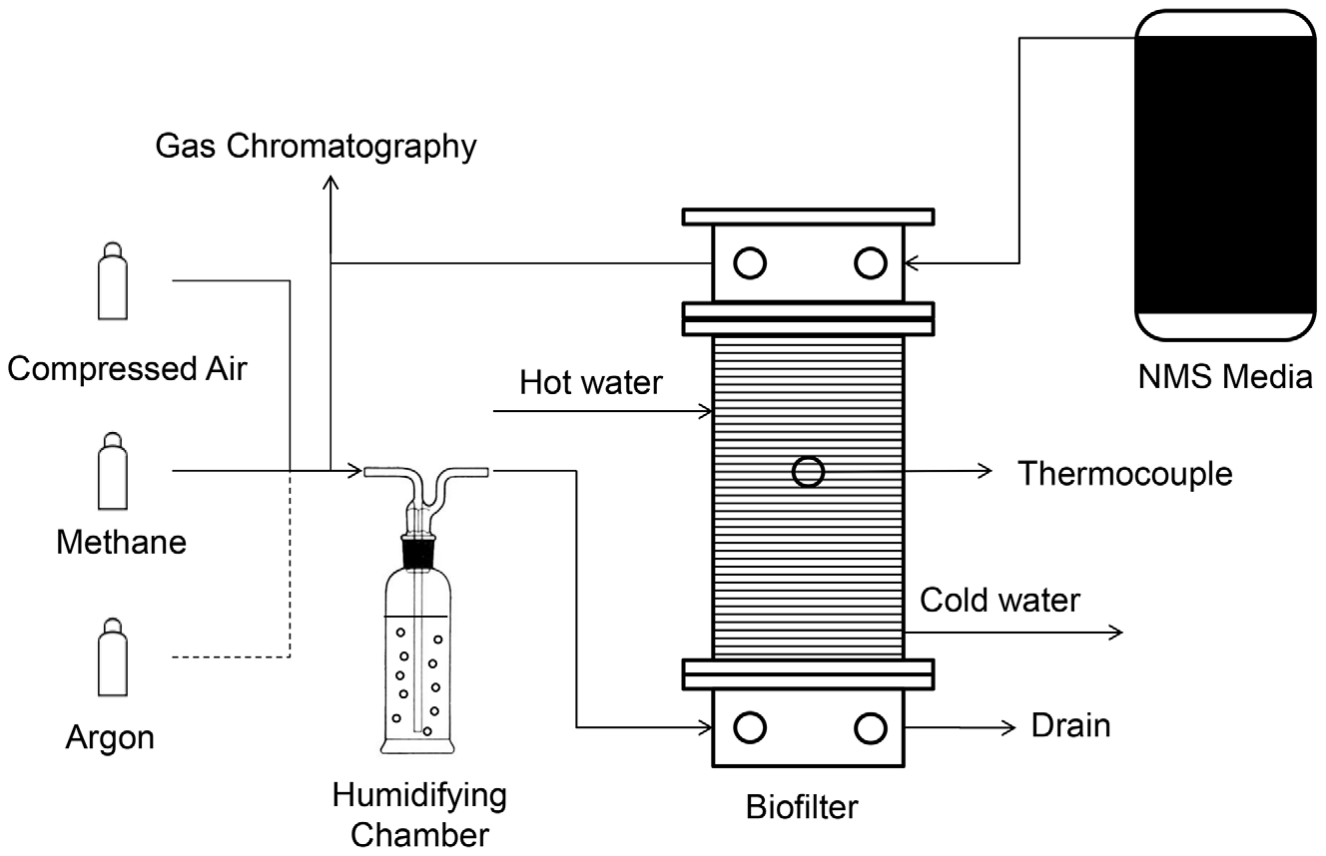
Schematic diagram of the laboratory-scale biofilter. Each biofilter comprised a central cylinder with an inner diameter of 15(i.e. above the bottom cap), a stainless steel screen (1 cm-mesh size) was inserted as a support for the coal bed. A thermocouple was inserted through the side of the cylinder for monitoring the temperature within the centre of the packing. The biofilter temperature was maintained by continuously circulating heated water through a tube coiled around the central component of the biofilter. The temperature of the heating water reservoir was adjusted so that the temperature within the biofilter was 30°C.

Two identical biofilters were designed and constructed from acrylic resin (Perspex) as detailed in Fig. 1. The two biofilters were packed with unsterilized coal to a bed height of 22 cm, equating to an empty bed volume of 3.89 L. The void volume within the coal bed was 0.39 L as determined by filling the airspace in the packing with water. Biofilter 1 was inoculated with a 100 ml of pure *M. sporium* culture mixed with 900 mL of NMS. Biofilter 2 was drenched with 1 L of sterile NMS alone (i.e. no *M. sporium*). Thus both biofilters contained microbes that naturally occurred on the coal packing and biofilter 1 additionally contained *M. sporium*. The coal beds were left to soak in their respective media for 24 hours after which the excess liquid was drained. A humidified methane and air gas stream (1% (v/v) methane in air at 0.2 L/min) was then continuously passed through each biofilter. Humidity was provided to prevent the coal bed from drying out. Sterile NMS medium (2 L) was added via the top of each biofilter every three days, the drain was opened after 10 min and the medium was then recirculated twice and drained again so that only a liquid film remained on the surfaces to feed the microbes.

Gas flow rates were periodically increased according to the values provided in Table I. Each flow rate increase was maintained until a stable methane conversion (defined as less than 5% variation of methane outlet concentration over a two week period) was achieved.

**Table I:** Gas flow rates, corresponding empty bed residence times (EBRT) and equivalent methane inlet loads used during biofilter experiments.

Gas flow rate (L/min)	EBRT* (min)	Methane Inlet Load (g/m^3^/h)
0.2	19.5	17.3
0.4	9.7	34.6
0.8	4.9	69.3
1.6	2.4	139
2.4	1.6	208

*EBRT was calculated by dividing the empty bed volume of the biofilter by the gas flow rate. The residence times based on the void volume in the coal bed are approximately 10 times shorter than EBRT.

### Gas analysis and determination of biofilter performance

Methane and carbon dioxide concentrations in the two biofilter inlet and exit streams were determined using a Shimadzu GC-8A gas chromatograph (GC) equipped with a thermal conductivity detector. Separation was achieved using an Alltech Hayesep DB 100/120 column with helium as the carrier gas. To assess whether methane removal originated from microbial action or system leakage, one hour before analysis argon (1% (v/v)) was introduced to the gas stream entering the biofilter as an inert internal standard. A cold trap was used to remove humidity from the gas stream prior to injection into the GC. Analyses were performed in triplicate every three days with concentrations determined relative to the internal argon standard based on peak areas. Biofilter performance was evaluated on the basis of empty bed volume to facilitate comparison with other studies. Methane inlet load (IL), methane removal efficiency (RE), methane elimination capacity (EC), and carbon dioxide production rate (PCO_2_) were calculated using the equations listed in Table II.

**Table II:** Parameters used to quantify biofilter performance.

Parameter	Method of determination*	Equation
Inlet load, IL (g m^−3^ h^−1^)	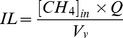	1
Methane removal efficiency, RE (%)	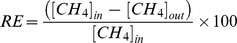	2
Methane elimination capacity, EC (g m^−3^ h^−1^)	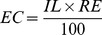	3
Carbon dioxide production rate, PCO_2_ (g m^−3^ h^−1^)	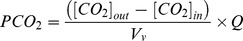	4

*The subscripts “in” and “out” indicate inlet and outlet concentration, respectively. [CH_4_], methane concentration (g m^−3^); [CO_2_], carbon dioxide concentration (g m^−3^); Q, gas flow rate (m^3^ h^−1^); V_v_, biofilter empty bed volume (3.89×10^−3^ m^3^).

### Microbial community analysis

The composition of the microbial community associated with the biofilter was analysed to identify microorganisms that were potentially involved in the removal of methane. For this, three coal pieces from each biofilter were collected during steady state at the highest gas flow rate (2.4 L min^−1^) and the total DNA of microorganisms associated with the coal was extracted [22]. Quality of the community DNA was checked by gel-electrophoresis and the DNA was then used to amplify the 16S rRNA gene, a marker gene that allows for the taxonomic and phylogenetic classification of microorganisms. The 16S rRNA gene was amplified by polymerase chain reaction (PCR) using the primers T7P-519F (5' TAATACGACTCACTATAGGG CAGCMGCCGCGGTAATWC) and M13R-926WR (5'CAGGAAACAGCTATGAC CCGYCAATTCCTTTRAGTTT) that target both bacterial and archaeal sequences [23]. PCR products were checked by gel-electrophoresis, purified and then amplified with a second round PCR primer to incorporate barcodes. These barcoded PCR products were then pooled and sequenced on a Titanium FLX pyrosequencer at the Ramaciotti Centre for Functional Gene Analysis at UNSW.

Pyrosequencing data in the form of flowgrams were processed using the MOTHUR software package version 1.31.1 [24]. Sequencing data were denoised, trimmed, quality-filtered and checked for chimeras following standard operating procedures [25]. Sequences were aligned against the bacterial reference alignment (14,956 sequences) from the SILVA database [26]. Sequences were clustered into an operational taxonomic unit at a 0.03 identity cut-off (roughly corresponding to species level cut-off) using the furthest neighbour algorithm. Representative sequences of operational taxonomic unit (OTU) at species level were then classified with the naive Bayesian rRNA classifier (version 2.5) using rRNA training set 9 of Ribosomal Database Project (RDP) [27]. Statistical comparison between the OTU counts in three replicates per biofilter was performed using Metastats [28], which employs false discovery rates for pairwise t-test comparisons to improve specificity in high-complexity environments, and separately handles sparsely-sampled OTU using Fisher's exact test. Metastas was run with 1000 iterations and p values <0.05 were considered significant.

## Results

### Methane removal

**Figure 2:**
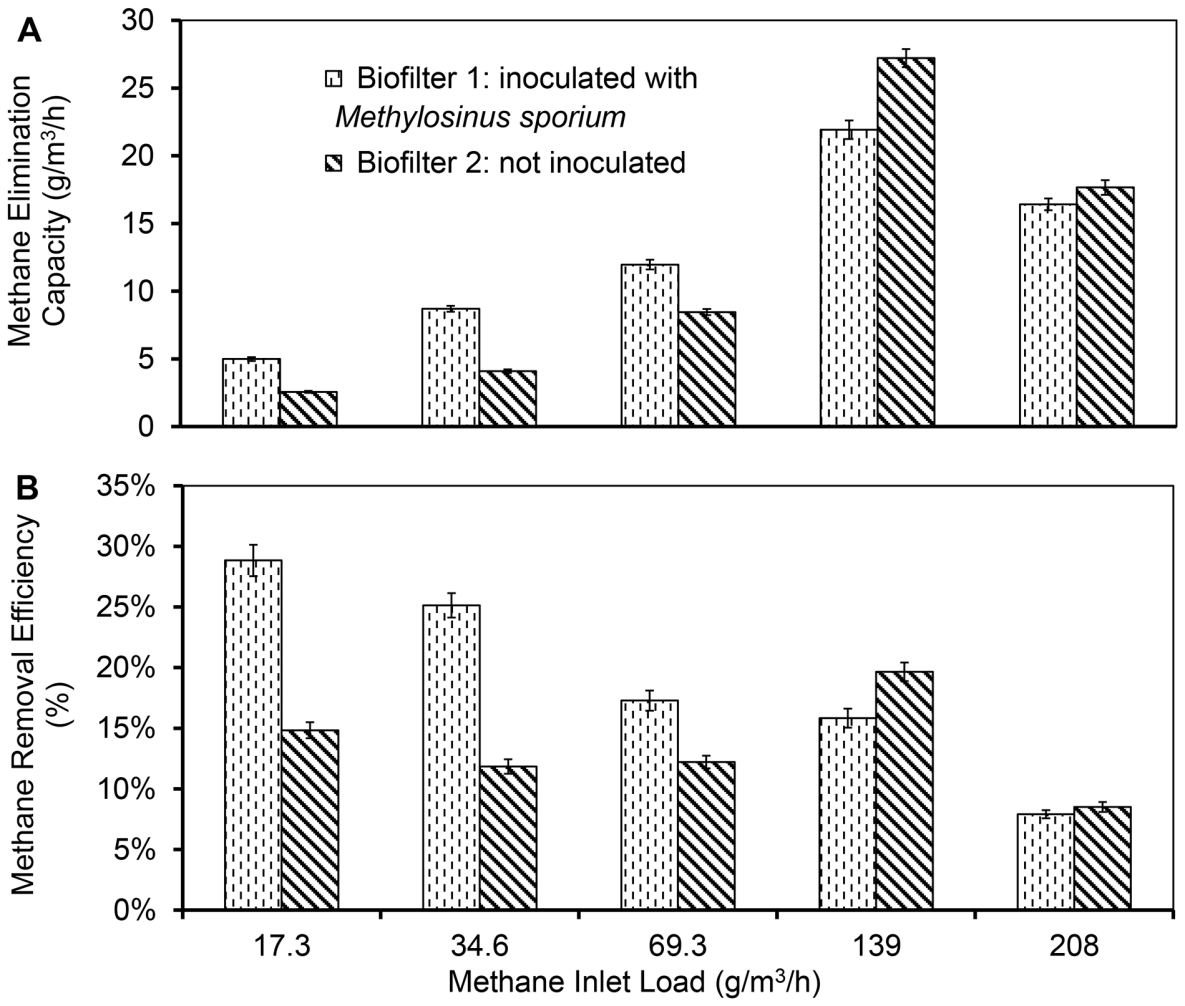
Effect of methane inlet load on the methane elimination capacity (A); and on the methane removal efficiency (B). Biofilters were operated at 1% methane (v/v) in humidified air; 30°C; non-sterilised coal as packing material. Each data point is the mean of eight measurements during two weeks of steady state operation, with error bars representing the standard deviation.

The effects of methane inlet load on the performance of biofilter 1 (inoculated with a pure culture of *M. sporium*) and biofilter 2 (non-inoculated) were investigated at 1.0% (v/v) methane in humidified air at increasing gas flow rates (indicated in Table I). Measurements of the internal argon standard demonstrated that there were no gas leaks in the system. At the initial flow rate of 0.2 L min^−1^, which is equivalent to a methane inlet load (IL) of 17.3 g m^−3^ h^−1^, steady state was reached after two weeks of operation. Fig. 2A illustrates the effect of increasing methane inlet load on the steady state methane elimination capacity (EC). In both biofilters, the EC increased with increasing ILs of up to 139 g m^−3^ h^−1^. When the IL was increased further, the EC decreased. The greatest rate of methane removal in the experiment was observed in the non-inoculated biofilter 2 at an IL of 139 g m^−3^ h^−1^, reaching 27.2±0.66 g methane m^−3^ empty bed h^−1^. The fact that the non-inoculated biofilter 2 eliminated significant amounts of methane indicates that the microorganisms native to the coal at the mine site may be catalytically active for methane removal. The inoculated biofilter 1 outperformed biofilter 2 at the three lower ILs during the initial 12 weeks. At the end of the experiment, the flow rate was returned to the starting value with an IL of 17.3 g m^−3^ h^−1^ and gas analysis three days later revealed that biofilter 1 and 2 operated at ECs of 5.20 and 2.70 g m^−3^ h^−1^, respectively. These ECs were within the same order of magnitude as those observed at the beginning of the experiment, inferring to comparable catalytic activity of the microbial communities at the beginning and end of the experiment.

Fig. 2B conveys these results as methane removal efficiency (RE), which is another important criterion for the potential environmental benefit of the biofilters. With the increasing methane inlet load, there was an overall trend of decreasing RE for biofilter 1 from 28.8±1.3% to 7.90±0.33%. The RE would have remained constant if the EC had increased by the same factor as the IL. The RE of biofilter 2 remained relatively low between 11.8±0.6% and 14.8±0.7% for ILs between 17.3 and 69.3 g m^−3^ h^−1^, then increased to 19.7±0.8% for an IL of 139 g m^−3^ h^−1^. When the IL was increased further to 208 g m^−3^ h^−1^, the RE dropped to 8.5±0.4%. The highest RE achieved under the experimental conditions was 28.8±1.3% at an IL of 17.3 g m^−3^ h^−1^ in biofilter 1, while at the highest EC in the experiment (biofilter 2 at 139 g m^−3^ h^−1^) the RE was 19.7±0.8%. Considering the additional cost of inoculation and the importance of elimination capacity at high inlet load (short residence time), it may be preferrable for field applications not to inoculate the coal bed.

### Carbon dioxide production

**Figure 3:**
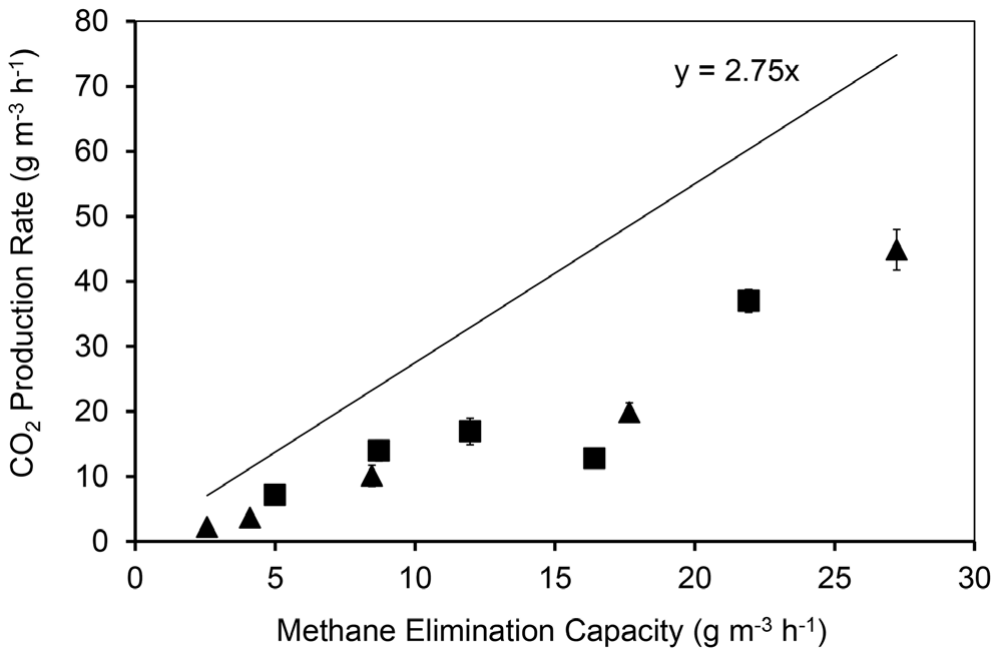
Carbon dioxide production rate (PCO_2_) in biofilter 1 (▪) and biofilter 2 (▴). Biofilters were operated at 1% methane (v/v) in humidified air; 30°C; non-sterilized coal as packing material. Biofilter 1 was inoculated with *M. sporium*. Each data point is the mean of eight measurements during two weeks of steady state operation, with error bars representing the standard deviation.

Methane consumed within the biofilters as shown in the previous section will be converted to biomass and/or carbon dioxide at the rate of the EC. Fig. 3 illustrates the relationship between EC and carbon dioxide production rate (PCO_2_). If all bioavailable methane was oxidised to carbon dioxide, the data points would fall on a line through the origin with a slope of 2.75 (based on the mass ratio of the two molecules). However, the actual data are below this line as some methane would have been used for producing microbial biomass. There is a general trend of increasing PCO_2_ with increasing EC in both biofilters and the degree of increase is similar for both biofilters. An exemption occurs at the highest IL (EC of 16.4 g m^−3^ h^−1^ in biofilter 1 and 17.7 g m^−3^ h^−1^ in biofilter 2) where PCO_2_ was lower relative to the general trend of increasing PCO_2_. This could mean that the highest load of methane per time favored the conversion of methane into biomass over the oxidation to carbon dioxide. The dry weight of the biomass was not directly measured because of its very low mass relative to the mass of coal to which the biomass was attached. Consequently, biomass production was estimated *via* the calculation of carbon mass balances as detailed in the next section.

### Carbon mass balance

By calculating carbon mass balances over biofilters 1 and 2 (Table III), the rate at which carbon accumulates in the biofilters (C_ACC_) can be estimated. During steady state, any physical adsorption of methane on the coal would be equal to the release of methane from the coal. Therefore the C_ACC_ is an indirect estimate for accumulated biomass. The values in Table III demonstrate that the estimated rate of carbon accumulation mostly increased with elevated IL in both biofilters. This indicates that biomass accumulation was faster at the higher ILs, possibly due to enhanced bioavailability of methane. The highest C_ACC_ was 9.51 g C m^−3^ h^−1^ in biofilter 1 at an IL of 208 g methane m^−3^ h^−1^. At an estimated carbon-to-dry-weight-ratio for bacteria of 50% [29], the accumulation rate would have been 19.0 g dry biomass m^−3^ h^−1^. Excess biomass accumulation could lead to clogging of the filter beds, however blockage was not observed over the duration of the experiment. Overall, the estimated values of carbon accumulation were in a similar dimension for both bioreactors. This indicates that inoculation with *M. sporium* was not required to achieve considerable microbial growth during biofilter operation.

**Table III:** Carbon mass balances and estimated carbon accumulation rates (C_ACC_) in the biofilters at varying methane inlet loads (IL).

	IL (g CH_4_ m^−3^ h^−1^)	C_IN_ (g C m^−3^ h^−1^)		C_OUT_ (g C m^−3^ h^−1^)		C_ACC_ (g C m^−3^ h^−1^)
		CH_4_	CO_2_	CH_4_	CO_2_	
Biofilter 1	17.3	13.0	0.23	9.24	2.18	1.81
	34.6	26.0	0.46	19.4	4.27	2.79
	69.3	51.9	0.93	43.0	5.55	4.28
	139	104	1.87	87.4	12.0	6.47
	208	156	2.78	143	6.27	9.51
Biofilter 2	17.3	13.0	0.23	11.1	0.84	1.29
	34.6	26.0	0.46	22.9	1.45	2.11
	69.3	51.9	0.93	45.6	3.69	3.54
	139	104	1.87	83.5	14.1	8.27
	208	156	2.78	143	8.19	7.59

Note: The carbon entering biofilter 1 and 2 (C_IN_) was the methane introduced to the system (CH_4_)_IN_ and the carbon dioxide in the inlet air (CO_2_)_IN_. The carbon introduced *via* methanol in the nutrient solution was less than 0.04 g C m^−3^ h^−1^ and was therefore neglected. The carbon exiting biofilter 1 and 2 (C_OUT_) consisted of the unconverted methane, (CH_4_)_OUT_ and the carbon dioxide leaving the system (CO_2_)_OUT_.

### Microbial communities on the biofilter coal packing

**Figure 4:**
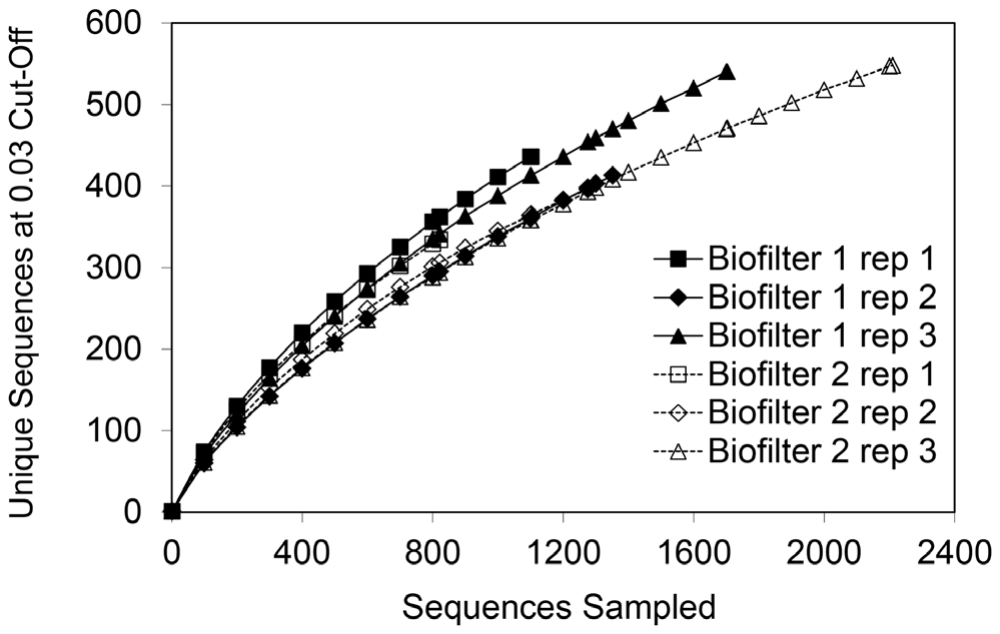
Rarefaction curve. Rarefaction curve of unique sequences at 0.03 sequence identity cut-off for the 16S rRNA gene of the microbial communities of three replicates for biofilter 1 and 2.

Analysis of the microbial communities at the end of the biofiltration experiments by pyrosequencing generated 8465 high-quality sequences of the 16S rRNA gene. Clustering of these sequences resulted in 1849 OTUs at “species level” (i.e. >97% sequence identity). Rarefaction analysis (see Fig. 4) showed no significant difference in the OTU richness among the three replicates of each biofilter, which indicates that the microbial communities had similar numbers of species. The shape of the rarefaction curve approached a line with increasing sampling effort, which shows that the sampling effort has been sufficient to capture the diversity within the communities associated with the biofilters.

The most abundant OTU (6.2+/−0.7% and 8+/−1.8% of all sequences of biofilter 1 and 2, respectively) could be assigned to the genus *Methylocystis*, a group of methanotrophic alpha-proteobacteria (type II methanotrophs) [30] (Table IV and Data S2). No other known methanotrophs could be identified in the dataset and the 16S rRNA gene of the inoculated *M. sporium* was also not detected (bioinformatic analysis showed that the PCR primers used had 100% match to the *M. sporium* sequence in the NCBI database). This indicates that the *Methylocystis* OTU is likely to have been the main organism responsible for biological methane removal in the biofilters at 140 days of operation. There was no significant difference in the abundance of this *Methylocystis* OTU between biofilter 1 and 2, which is consistent with the similar performance of the biofilters at the end of the experiment (Fig. 2).

**Table IV:** Mean abundance of OTUs in biofilter 1 and 2 and their taxonomic assignment.

Biofilter 1		Biofilter 2					
Mean	Stderr	Mean	Stderr	p value	Taxa	Level	Confidence
0.062	0.007	0.080	0.018	0.604	*Methylocystis*	genus	0.97
0.030	0.011	0.102	0.024	0.024	*Luteimonas*	genus	1
0.075	0.058	0.004	0.004	0.206	*Streptacidiphilus*	genus	0.54
0.034	0.013	0.021	0.009	0.647	Acidobacteria_Gp10	subdivision	0.97
0.042	0.011	0.009	0.009	0.037	*Sediminibacterium*	genus	0.59
0.002	0.001	0.037	0.009	0.011	Bacteria	domain	0.93
0.025	0.004	0.016	0.002	0.039	*Filomicrobium*	genus	0.51
0.019	0.006	0.004	0.004	0.038	*Anaerolineaceae*	family	0.67
0.004	0.002	0.022	0.007	0.029	*Ohtaekwangia*	genus	0.99
0.000	0.000	0.020	0.006	0.015	Acidobacteria_Gp10	subdivision	0.96
0.015	0.005	0.007	0.002	0.162	*Nocardioides*	genus	0.92
0.017	0.003	0.006	0.002	0.022	*Hyphomicrobiaceae*	family	0.79
0.001	0.001	0.017	0.006	0.028	*Anaerolineaceae*	family	0.55
0.009	0.003	0.004	0.003	0.229	*Ohtaekwangia*	genus	0.56
0.010	0.006	0.004	0.001	0.588	*Hydrogenophaga*	genus	0.74
0.004	0.002	0.012	0.004	0.050	*Nocardioides*	genus	0.93
0.014	0.011	0.001	0.001	0.222	Bacteria	domain	0.97
0.004	0.001	0.011	0.003	0.037	*Microbacteriaceae*	family	0.95
0.000	0.000	0.014	0.003	0.005	Pseudoxanthomonas	genus	1
0.004	0.001	0.008	0.005	0.661	*Nocardioides*	genus	0.96
0.009	0.004	0.003	0.002	0.146	*Actinophytocola*	genus	0.93
0.002	0.000	0.009	0.004	0.097	*Conexibacter*	genus	0.73
0.005	0.001	0.006	0.003	0.956	*Nocardioides*	genus	0.97
0.008	0.003	0.003	0.001	0.176	Rhizobiales	order	0.77
0.000	0.000	0.008	0.004	0.065	Chloroflexi	phylum	0.6

Note: Only OTUs with an average abundance of at least 0.5% across all samples are shown. P values of Metastats analysis are shown and are considered significant when <0.05. Confidence refers to RDP classification confidence. Assignments are shown for the lowest taxonomic level with >0.5 confidence.

In addition to this methanotroph, the biofilters harboured taxonomically diverse communities of bacteria. The second most abundant OTU (6.8% of all sequences) could be assigned to *Luteimonas*, a genus comprising aerobic chemoorganotrophs with a wide substrate range [31]. Chemoorganotrophs are organisms that oxidise organic compounds as their energy source [32]. Other chemoorganotrophs (with greater than 0.5% frequency) with no known ability to utilise methane belong to the genera *Sediminibacterium*, *Nocardioldes*, *Conexibacter* and *Actionphytocola*. Other chemoorganotrophs, such as *Filomicrobium* and *Pseudoxanthomonas*, found on the biofilters have been previously associated with the degradation of hydrocarbons or aromatics [33], [34] (Table IV). It is likely that those chemoorganotrophs derived energy from decomposition of biomass generated from methane oxidation or directly from the bioavailable compounds of the coal. Some of these chemoorganotrophs also showed statistically-significant differences in their abundance between biofilters (e.g. *Sediminibacterium* and *Filomicrobium*), which might reflect different substrate availability between the two biofilters.

Other nutrient input into the biofilters' microbial communities could also come from the genus *Azohydromonas* (0.33% abundance, see Data S2), whose members can fix nitrogen [35].

## Discussion

The current study highlights the potential application of coal-packed biofilters for partial methane removal from coal MVA. Increasing the methane IL by raising flow rates at a constant low methane concentration representative of MVA increased the methane EC to an optimal level after which EC values decreased (Fig. 2). Similar trends have been observed in other biofiltration systems where further flow rate increases saw EC values either be maintained [36], [37] or dropping [17].

Several physical factors may affect the bioavailability of methane. Firstly, the movement of methane molecules within the gas phase plays a role. The Reynolds number remained well below 10 for all tested flow rates, which indicates laminar flow. Therefore, the movement of methane molecules within the gas towards the water surface was not assisted by turbulence, but governed by convection and diffusion. Secondly, interfacial transfer from the gas to the water phase may be a limiting factor. As methane is poorly soluble in water (0.022 g of methane/kg of water), the driving force for methane uptake is low. Consequently, methane diffusion into the liquid phase is slow [38]. As the empty bed residence time (EBRT) decreases with increasing gas flow rate, the time available for methane transfer across the gas/liquid interface is shorter and in turn, restricting methane availability to the microorgasnisms [39] and decreasing RE [17]. On the other hand, the increasing gas flow rate might increase the rate of diffusion of methane from the gas phase into the liquid phase. This is because an increasing IL replenishes used methane faster and therefore increases the methane concentration gradient across the interface [40]. Such effect may have contributed to the increasing EC with increasing IL up to 139 g m^−3^h^−1^. Thirdly, the distribution of methane that has entered the liquid phase would be governed by methane diffusion through the water and extracellular polymeric matrix of the microorganisms as well as by cellular methane uptake and oxidation. It is therefore likely that an increase in the thickness of the microbial community will decrease the availability of methane to the deepest layer of cells on the coal surface. At this stage however, it remains unclear which of the above-mentioned factors is the most limiting for the biofilter performance.

The change of the oxidative capacity of the microbial community over time is unknown and may overlay the effects of changing ILs. It is known however, that the final return of the flow rate to the initial value revealed catalytic activities of the microbial community at the beginning and end within the same order of magnitude. Biological factors that influence the oxidative capacity of the biofilter include biomass quantity and the bio-catalytic activity of the biomass. Increasing biomass quantity as indicated by the carbon accumulation rate in Table III, will only be beneficial until all coal surface area is covered with microbial community up to a thickness that allows efficient methane diffusion to the cells. Maximising the packing surface area for microorganisms in order to increase the EC may be achieved by using smaller coal pieces or high-porosity coal; however this could result in increased back pressure and eventually in blockage of the biofilter.

In addition to the quantity of the microbial biomass, its bio-catalytic activity is important for biofilter performance. The biological methane oxidising activity is determined by the community composition of microorganisms and their relevant enzymes as well as by the bio-available methane concentration. The community analysis illustrated that in both biofilters a large proportion of the microbial diversity found had no known capacity for methane oxidation and this may provide an opportunity for optimisation. The primary difference between biofilter 1 and 2 was the initial inoculation of biofilter 1 with the methanotrophic *M. sporium*. This addition of *M. sporium* to the naturally occurring microbial community may have directly or indirectly caused the initial higher EC and RE values in biofilter 1 compared to biofilter 2. At the highest IL, performance of the biofilters became comparable and the methanotroph *Methylocystis sp.* was dominant in both biofilters. Interestingly, no *M. sporium* was detected. It is not known at what point *Methylocystis sp.* began to dominate in the biofilters and when *M. sporium* disappeared in biofilter 1. Several strains of *Methylocystis sp.* are known for their high affinity for methane and the ability to oxidise methane even at low atmospheric concentrations [41]. Reported methane monooxygenases of the genus *Methylocystis* have lower Km values (3.2–4 µM) than those of the genus *Methylosinus* (8.3–62 µM) [42]. Since the Km value signifies the methane concentration at which the enzyme reaches half-maximal rate, *Methylocystis sp*. might be better adapted to low methane concentrations than *M. sporium*, however these values may vary at the strain level. While all Km values are well below the solubility of methane in water (1375 µM), mass transfer limitations might have led to a much lower available methane concentration in contact with the enzymes so that Km values may play a role in biofilter performance.

A unique element of the biofilters investigated in this work was the use of coal as the support medium and microorganisms inherent to the coal surface as the methane oxidisers. Other studies have used alternative supports with or without inoculation to treat gas streams containing low methane levels. However, some of their biofilters required a lengthy EBRT to treat 1% methane (v/v) in the air inlet stream. For instance a composted pine bark biofilter tested by du Plessis et al. (2003) needed a residence time of 20 minutes to remove 40% of the methane. Some studies that investigated residence times more similar to the present study are listed in Table V. The comparison indicates that the performance of the biofilters in the present study was within the range of EC and RE of alternative systems.

**Table V:** Comparison of biofilter performance in the present work with biofiltration results from other studies at similar empty bed residence times (EBTR).

Reference	[CH_4_] (% (v/v) in air)	IL (g/m^3^/h)	EBRT (min)	Packing Material	Inoculation	CH_4_ EC (g/m3/h)	CH_4_ RE (%)
[16]	1.0	77	5	Glass tubes	Yes	15.4	20
[9]	0.70–0.75	71	4.3	Mature compost	No	13.5	19
[9]	0.70–0.75	71	4.3	Inorganic material	Yes	29	41
[17]	1.0	128	3.2	Gravel	Yes	45	35
[13]	0.03–0.43	28	4.2	Gravel	Yes	12±0.20	43±0.60
Present study B1*	1.0	139	2.4	Coal	Yes	22±0.68	16±0.79
Present study B2*	1.0	139	2.4	Coal	No	27±0.66	20±0.77

*B1 corresponds to biofilter 1; B2 corresponds to biofilter 2.

The data from Table V provides an indication of biofilter performance in terms of RE and EC, but the implications in regards to biofilter footprint also need to be considered when investigating feasibility. The highest EC in this study (27.2 g methane m^−3^ empty bed h^−1^) at a RE of 20% was achieved with an EBRT of 2.4 min in biofilter 2, which corresponds to a theoretical biofilter size of 7,200 m^3^ for an MVA gas flow rate of 50 m^3^ s^−1^. Although this is physically large, there is almost no infrastructure requirement; a reactor enclosure is not required, the bed material of coal is readily available on site, and no inoculation is needed. The only critical components are distributing the inlet flow across the bed and keeping the bed moist with a nutrient solution. With a height of about 4.5 m, a footprint of 40×40 m would give sufficient residence time for 50 m^3^ s^−1^ MVA based on the non-optimised results from our study. Even at a RE of 20%, the absolute emission reduction would be large due to the scale of the emissions. Compared to previous suggestions for biofilter control of methane in MVA, this is the first potentially practical design, and is inexpensive. The required residence time and therefore the footprint of the biofilter may be decreased by improving the mass transfer of methane to the microorganisms. One reported approach is the introduction of a biocompatible organic solvent to improve the gas-liquid interfacial mass transfer rate of methane [43], [44]. This potential improvement may increase or decrease biofilter operating costs indicating a cost/benefit analysis would be required to assess the feasibility of such a strategy [45].

In conclusion, methane biofiltration technology could offer partial methane removal from MVA at low capital and operating costs. The present study demonstrates that, in principle, coal can be used as a low cost packing material, which does not require inoculation to achieve ECs comparable to those reported for other support materials with inoculation. However, the required residence time will need to be shortened to accommodate MVA gas flow rates of higher than 50 m^3^ s^−1^ in biofilters of reasonable sizes. A possible future strategy for such improvement is the investigation of conditions that favour the growth of methanotrophs over other organisms in a non-sterile environment. Another strategy would be an engineering approach to improve the transfer of methane from the inlet stream to the microorganisms.

## Supporting Information

Data S1 **Coal characteristics.**
(DOCX)

Data S2 **Relative overall abundance of operational taxonomic units (OTU) obtained from coal samples of biofilter 1 and 2.** Only OTUs with a relaltive overall abundance of >0.1% are shown.
(XLSX)
