# Supplementary material for: Coal-Packed Methane Biofilter for Mitigation of Green House Gas Emissions from Coal Mine Ventilation Air
Source: PLoS One. 2014 Apr 17;9(4):e94641. doi: 10.1371/journal.pone.0094641 (PMC3990720; doi:10.1371/journal.pone.0094641)
Supplement: Data S1 — Coal characteristics. (DOCX) [file pone.0094641.s001.docx]

**Coal Characteristics**

The water holding capacity of the coal packing material was 3.1 x 10^-2^ g/g dry coal as determined by soaking air-dried coal samples in distilled water for 4 hours at 22˚C and measuring the loss of weight achieved after drying at 110˚C to a constant weight (~3 hours) (Kaji et al. 1984). The water holding capacity is representative of the amount of nutrient solution held by the coal surfaces to feed the microorganisms.

The moisture content of the coal was 2.5% and the ash content was 11.2% which were evaluated according to procedures detailed in Australian Standards AS 2434.1-1999 and AS 1038.10.4-2001, respectively (Commitee of Standards Australia 1999, 2001).

**References**

Kaji R, Muranaka Y, Otsuka K, & Hishinuma Y (1984). Water absorption by coals: effects of pore structure and surface oxygen. Fuel*,* 65, 288 - 291.

Commitee of Standards Australia (1999). AS 2434.1: Method for the analysis and testing of lower rank coal and its chars. *Part 1: Determination of the total moisture content of lower rank coal* (pp. 3-11). Sydney, Australia: Standards Australia International.

Commitee of Standards Australia (2001). AS 1038.10.4: Coal and coke-Analysis and testing. *Part 10.4: Determination of trace elements-Coal, coke and fly-ash-Determination of fluorine content-Pyrohydrolysis method* (pp. 3-14). Sydney, Australia: Standards Australia International.
